# On Some Disorders of the Nervous System in Childhood

**Published:** 1871

**Authors:** Charles West

**Affiliations:** Physician to the Hospital for Sick Children


*On some Disorders of the Nervous System in Childhood.* By CHARLES WEST, M. D., Physician to the Hospital for Sick Children.

LECTURE I.

After a short sketch of the founders of the lectures, Dr. West observed that the diseases of early life differ in many important respects from those of adults; they run a different course; symptoms have a different import; and the probabilities of death or recovery are determined by different considerations.

Diseases of the nervous system present in childhood peculiarities more remarkable than those of any other class. This is owing to the circumstance that the nervous system is more unformed; its functions more rudimentary; its condition one of chance and development, the like of which does not take place in organs of respiration and circulation, or even digestion. Among adults a large class of ailments consist of simple pain, independent of local disease. In infancy and childhood, on the other hand, pain referred to any part signifies, almost without exception, that disease of some sort is going on there or near at hand. Even in later childhood, the rarity of real neuralgia is extreme. In two classes of cases this caution is important—first, in pains referred to the head, and, secondly, in pain referred to the leg. The former nearly always indicate organic disease, especially tumor of the brain and tubercular hydrocephalus; the latter usually is due to hip-joint disease. Pain dependent on cerebral disease is rarely limited to one part of the head, in neuralgia it is commonly localised. One form of neuralgia is not very uncommon in childhood, that known in later life as "sick headache," but Dr West has never met with it in children who had not gone into the schoolroom, and often found it to be distinctly due to too continuous mental application.

Disorder of motor power is frequent in childhood, though pain is rare. Derangements of digestion, too long fastings, a too full meal, the irritation of teeth, the outbreak of an eruptive fever, all may give rise to convulsive movements or a fit of convulsions. The first convulsions in childhood are generally due to eccentric causes, or to some primary disease of brain; but, in the latter case, are nearly always preceded by other symptoms (though it may be that these are obscure,) and are accompanied by other grave evidences of nervous disease, especially by local weakness, inequality of pupils, &c. Convulsive movements in early life are often partial, but such attacks have always a great tendency to become general. In infancy and early childhood the convulsions are commonly violent, attended with unconsciousness, and death not unfrequently takes place in the paroxysm. They have a great tendency to recur without obvious exciting cause, and then receive the name of epilepsy. In proportion to the frequency of the fits, the mental development is retarded. Tne same class of causes which produce epilepsy in the child tend, later on, to cause chorea, a disease which may, however, occur at any age. Dr. West has seen one case at eight months of age. Whatever may have been their original cause, all convulsions show a great tendency to recur. Out of forty-two epileptic children, not including epileptic idiots, twenty-three had suffered from fits in infancy. Hereditary predisposition was observed in only seven out of the forty-two, and in three only of the twenty-three. Hereditary tendency to epilepsy probably comes into play later in life than the age of childhood, just as the hereditary tendency to hysteria manifests itself with the evolution of the sexual system, and, still later, that to insanity under the pressure of life's cares. Those cases of fits in childhood are more likely to result in permanent epilepsy, in which no exciting cause is discoverable, and in which the causes have acted on the emotions and higher intellectual powers. Frequent attacks of *le petit mal* are of grave augury, and are very likely to be overlooked in the child. Fits are sometimes accompanied by moral perversion, a condition which is often associatied with mental dullness, but in the child shows itself first, and is the more easily recognised, while in later life the reverse is the case. In the treatment of convulsions of childhood, intestinal derangements must be carefully set right. Worms, Dr. West thinks, are a less frequent cause than they are assumed to be by the public, lay and medical. All the alleged specific remedies for epilepsy have, in Dr. West's hands, failed more or less completely. The only one exerting any influence is the bromide of potass, which in a few cases has given remarkable results; but in the majority of instances, the improvement, often manifested at first, is not maintained; the agent loses its effect altogether, or has to be augmented until doses are reached which cause so much depression that the remedy has to be discontinued. Lastly, moral treatment may do a good deal towards curing epilepsy. To this Dr. West ascribes the cessation of fits so often witnessed during a sojourn in a hospital.—*Lancet.*

